# Metrics for biodiversity and health policy integration

**DOI:** 10.1371/journal.pgph.0004624

**Published:** 2025-07-01

**Authors:** David Nogués-Bravo, Sarah Whitmee, Liz Willetts

**Affiliations:** 1 Center for Macroecology, Evolution and Climate, Globe Institute, Faculty of Health and Medical Sciences, University of Copenhagen, Copenhagen, Denmark; 2 Centre on Climate Change and Planetary Health, London School of Hygiene and Tropical Medicine, London, United Kingdom; 3 Inter-American Institute for Global Change Research, Clayton, Panama; Georgetown University, UNITED STATES OF AMERICA

## Abstract

Despite over a decade of progressive commitments from parties to the Convention on Biological Diversity (CBD), integrated biodiversity and health indicators and monitoring mechanisms remain limited, hampering achievement of the sustainable development goals and improvements in health and well-being. Adoption of the Kunming-Montreal Global Biodiversity Framework (2022) and the Global Action Plan on Biodiversity and Health (2024) provide a renewed entry point to shape the way governments approach health and wellbeing and address the environmental burden of disease. This is a critical opportunity that scholars at the health-environment nexus should not miss. This Perspective outlines building blocks to mobilize the field, starting with essential terminology and a scope of metrics needed by governments. We then evaluate elements to be considered in the construction of integrated metrics, including concepts, overarching challenges, a review of scientific hypotheses from an ecological perspective, as well as a set of principles and characteristics for indicators. To raise awareness across parallel communities of practice working at the health-environment nexus, we then briefly examine four approaches to integrated metrics developed by: conservationists, Indigenous scholars, One Health experts, and planetary health experts. We conclude with actionable steps to enhance governance, mobilize funding, and apply integrated indicators in national and global strategies. A broad science community is needed to support national governments to meet global commitments to address biodiversity loss and the environmental burden of disease concurrently. The overall aim of this paper is to contribute to addressing biodiversity loss by effectively linking policy and transdisciplinary practice at the health-environment nexus.

## Introduction

**Fig 1:**
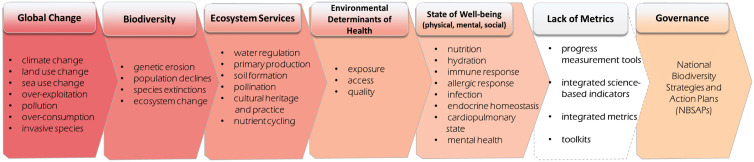
Communicating the Relationship Between Biodiversity and Ecosystems and Human Health into National Governance The accelerating magnitude and pace of global change drivers are leading to significant biodiversity decline. This decline reduces the capacity of ecosystems to perform essential functions and to sustain critical ecosystem services that support human health. The absence of integrated metrics undermines our ability to govern, monitor and evaluate implemented actions and interventions that sustain and promote both biodiversity and health. Metrics are needed to inform national planning including national biodiversity strategies and action plans.

An integrated approach to sustain and improve both biodiversity and human health is vital to sustainable development and well-being [1]. But national and multilateral commitments on joint indicators and monitoring mechanisms for biodiversity and health continue to stall. This limits policy progress and undermines conservation and public health efforts. See Fig 1.

Explicit demand from national governments on what is needed to monitor, evaluate, and implement integrated governance on biodiversity and health exists. For more than a decade in global decisions under the Convention on Biological Diversity (CBD), nearly 200 countries have sequentially requested biodiversity and health i) “progress measurement tools”, ii) “integrated science-based indicators”, iii) “integrated metrics”, and iv) “toolkits” compiling these assessment tools [2–8]. See S1 Table. While limited financial resources may partly explain why linked biodiversity-health indicators have not advanced inside the CBD, indicators also failed to advance significantly in wider science communities of practice or in international fora concerned with biodiversity or human health science [9–12] and this is true for qualitative, quantitative, and science-based measures. This gap exists despite extensive literature on conceptual frameworks on the links between biodiversity and health [12–37] and ongoing programmes of work on aspects of ecosystem and public health worldwide across UN institutions and international and national organizations. The divide between intergovernmental calls for information, made on consensus, and the limited response in the scientific community is the gap we aim to address in this paper.

In this context, greater capacity is needed to understand the barriers to advancing the science and policy implementation of integrated metrics. This paper provides an overview on the science of integrated metrics from ecological and health perspectives and then reviews how leading holistic policy approaches seek to monitor the governance of biodiversity and health. This paper is a transdisciplinary effort uniting ecology, medicine, public health, and environmental governance expertise. A goal of this work is to, in turn, mobilize and strengthen coordinated action and investment across science, policy, and law communities to improve integrated ecosystem and public health management. This paper is intended to build capacity for the community of nexus scholars working to address integrated metrics. Important terms and conceptual building blocks for integrating knowledge are in Box 1.

Developing shared metrics can facilitate the integration of health into biodiversity strategies and biodiversity into health and other strategies concurrently. A structured method for quantifying, valuing, monitoring, and mitigating the impacts of biodiversity loss on human health—ideally from local to national and global scales—will elevate biodiversity conservation as a priority for government funding. Biodiversity conservation measures are more likely to gain traction with decision-makers if they are conveyed as also impacting wider determinants of human health. While the lack of standardized metrics slows progress, weak indicators hinder policy enforcement, funding, and monitoring, and environmental management and protection [38].

Improving and applying integrated metrics on biodiversity and health ensures that environmental determinants of health are recognized at the national level and that efforts are made to track the environmental burden of disease. Historically, integrated indicators for biodiversity have fallen under the governance domain of multilateral environmental agreements, as opposed to global health ones [39]. Several strategies are available where integrated metrics, once developed, could immediately be applied at the global and national levels, including implementation of the CBD’s Kunming-Montreal Global Biodiversity Framework and its Monitoring Framework. Governments are currently working to update their National Biodiversity Strategies and Action Plans (NBSAPs). Integrated metrics on biodiversity and health interlinkages can be embedded in an NBSAP to shape and inform the way countries consider, address, invest in, and strategize approaches to the environment-health nexus.

**Box 1: Biodiversity and Health Terminology Explainer**

**Biodiversity** is the foundation of ecosystem functioning and services and is measured by the diversity of genetic, species and ecosystem diversity. Under the Convention on Biological Diversity, **biological diversity**, includes variability among living organisms from all sources including, inter alia, terrestrial, marine and other aquatic ecosystems and the ecological complexes of which they are part; this includes diversity within species, between species and of ecosystems [40]. Therefore, the diversity between ecosystems and within ecosystems contribute to biological diversity.

**Ecosystem integrity** refers to a measure of ecosystem structure, function, and composition relative to the reference state of these components being predominantly determined by the extant climatic–geophysical environment [41].

**Ecosystem services** refer to the benefits that humans receive from natural ecosystems. These services are essential for human survival and well-being [42].

**Environmental determinants of health** are all the non-medical, environmental factors that influence health outcomes. They include the state and conditions of the natural environment where people are born, grow, work, live, and age, as well as influences from natural or human induced environmental change such as soil degradation or ocean acidification.

**Human health** is the state of complete physical, mental and social well-being and not merely the absence of disease or infirmity, [43] while **public health** is the science and art of preventing disease, prolonging life, and promoting health through the organised efforts of society [44].

**Integrated Science-Based Metrics** are comprehensive measures that combine data and insights from multiple scientific disciplines to assess complex issues holistically. These metrics integrate ecological, health, and socio-economic data to provide a nuanced understanding of the interplay between systems such as biodiversity and human health. Designed for policy relevance, they support informed decision-making by offering scalable, evidence-based insights that reflect real-world conditions and trends. These metrics can quantify the role of nature as a determinant of health and describe causal links between the state of biodiversity upon which natural systems and human health depend.

The **One Health Approach** is “an integrated, unifying approach that aims to sustainably balance and optimize the health of humans, animals, plants and ecosystems. It recognizes the health of humans, domestic and wild animals, plants and the wider environment (including ecosystems) are closely linked and interdependent. The approach mobilizes multiple sectors, disciplines and communities at varying levels of society to work together to foster well-being and tackle threats to health and ecosystems, while addressing the collective need for clean water, energy and air, safe and nutritious food, taking action on climate change, and contributing to sustainable development” [28].

**Planetary Health** refers to the health of human civilisation and the state of the natural systems on which it depends [16,45].

### Scope for approaching integrated science-based indicators

Monitoring mechanisms are key to policy implementation, ensuring accountability, steering investment, and to effective evaluation and policy improvements. Three tiers of metrics are generally employed in intergovernmental decisions and embedded commitments. First, *qualitative progress measures* that provide information on the recognition or application of a concept in planning, strategy, or budgeting. This could be reported subjectively based on written observation of attitudes, willingness, or demand; or it could be reported numerically, e.g., the number of municipalities that recognize the human right to a clean, healthy, and sustainable environment. Second, *quantitative measures*, such as the calculation of the proportion of households with access to potable water during a prolonged drought. Third, there are *integrated science-based metrics* that estimate a finding based on combining several variables, such as environmental burden of disease, which is based on comparative risk assessment of a range of environmental factors and expressed in terms of attributable deaths or disability-adjusted life years (DALYs) [46].

In national policy settings, it is useful to have all three types of metrics to gauge progress and trends in different ways and timescales, and at different phases of implementation. Often, countries who are the least resource equipped financially, technically, and technologically, or who are dealing with conflict or crisis, may only be able to report on qualitative progress measurement. A tiered approach to metrics enables all countries to participate flexibly, and for some countries to report comprehensively. While all three types of metrics are needed and requested by governments on biodiversity and health, integrated science-based metrics most closely link ecosystem management to public health and are where greater attention is needed. This section discusses science-based metrics in four ways: conceptual foundations, overarching challenges, scientific hypotheses, and principles and characteristics.

### Integrated metrics: Conceptual foundations

Integrated biodiversity and health metrics cannot advance without a rethinking of the conceptual and disciplinary frameworks that have historically kept these fields apart. Despite recent progress to link public health and the state of biodiversity [23], public health research and biodiversity science have evolved as distinct fields, each with its own set of methodologies, priorities, and terminologies. Public health has primarily focused on understanding and managing human health and diseases (i.e., negative impacts), emphasizing medical, social, and behavioral determinants. Conversely, biodiversity science has concentrated on the study of ecosystems, species, and genetic diversity, aiming to preserve and understand the diversity of life on Earth [47].

In a legal analysis of the Sustainable Development Goals (SDGs), Lajaunie and Morand (2021) concluded: “Biodiversity and ecosystem functioning are kept at the margin of health issues and not acknowledged as an integral part of the reflection on the improvement of health worldwide” [48]. Gittleman (2024) came to a similar conclusion in his environmental analysis of One Health and planetary health in which he proposed “retraining the current 10 million to 15 million medical practitioners worldwide to recognize ecological patterns associated with medical problems” [49].

This disciplinary separation contributes to a persistent neglect of the interconnectedness between biodiversity and human health [50]. As a result, opportunities for collaborative research, integrated data collection and analysis are undeveloped. Bridging these silos requires a concerted effort to develop interdisciplinary frameworks that recognize the interdependencies between human health and biodiversity and institutional structures that support this work. This may include creating shared platforms for data exchange, fostering cross-disciplinary training programs, and encouraging joint initiatives that bring together public health experts, ecologists, and policymakers, such as the U.S. National Academies of Science, Engineering, and Medicine workshop on Integrating Public and Ecosystem Health Systems to Foster Resilience in 2022 [51].

Beyond disciplinary divides, conceptual gaps also remain. Uptake of culturally-sensitive planetary and ecosystem-based perspectives is also important to resolving conceptual dissonance [50]. Indigenous knowledge systems offer relational understandings of health and nature that challenge dominant scientific frameworks and point to alternative ways of measuring interconnection. Therefore, metrics that align with Indigenous perspectives are needed to effectively measure and understand health and well-being.

Biodiversity and health interlinkages are clearly an element to ecological and social resilience and this idea should or could also be considered in the development of metrics. In the last decade, the downward trend of biodiversity loss has accelerated to nearly 2 percent decline per year across terrestrial and marine ecosystems, and 4 percent per year in freshwater ecosystems [38,52,]. The planetary boundaries for genetic and functional biosphere integrity have been transgressed [53]. And biodiversity loss, ecosystem collapse, and natural resource shortage top the outlook of the 2024 global risk insights report of the World Economic Forum [54].

### Integrated metrics: Common challenges for indicators and their application

Developing indicators and metrics based on scientific evidence for the biodiversity-health nexus is a daunting task. Environmental experts flag some key gaps. Niemeijer and de Groot (2008) described the general development and selection process of environmental indicators as not sufficiently systematic nor transparent [55]. The Intergovernmental Science-Policy Platform on Biodiversity and Ecosystem Services (IPBES) (2019) recognized the lack of ecological metrics that can translate health risks or health gains [19]. Experts on planetary boundaries highlight difficulties in identifying adequate control variables as well as the need for several control variables to monitor specific natural systems [56]. GEO BON points out several challenges of indicator development for ecosystem services, including that they are highly context-specific to ecosystems, cultures and socio-ecological systems, hard to aggregated across scales, and require participatory approaches to select priority variables for policymaking [57]. Crucially, GEO BON emphasizes the complexity of choosing a single variable to measure each aspect of an ecosystem service that may have multiple values depending on perspective [57]. Planetary health scholars highlight barriers related to difference in methodologies and priorities for different elements of biodiversity [58]. Reis et al (2015) lament how tools developed to address proximal environmental health issues are limited in scale and temporal scope when faced with planetary boundaries and planetary health risks [37].

Scholars from related fields identify common challenges that are relevant to the development of biodiversity and health indicators. Climate and health literature demonstrates the difficulty in scaling down monitoring on climate-sensitive morbidity and mortality from global to national or local contexts, largely due to data scarcity [59]. Even in data rich countries, feasible climate-health data may be lacking or there may be additional data processing required [60,61]. Efforts among transdisciplinary scholars to bring human health into ecological frameworks or ecological indicators into public health frameworks underline how correlation between health improvement and an environmental aspect is not always followed by positive or significant evidence or behavior change [62,63]. A range of literature on One Health portrays challenges to assessing science-policy coordination for indicators, including collaborating between a wide diversity of stakeholders (e.g., political fragmentation, trust, time consuming effort, competition) [64], ensuring indicators reflect balanced representation of different sectors [65], and the overall paucity of evaluation methodology on One Health effectiveness. [64,66]. There is also disproportionate research attention on biomedical versus ecological aspects of spillover, but where done there is also an inherent difficulty with time delays between a triggering event and evidence of human health outcome [66,67]. Finally, there is uncertainty that positive outcome data (i.e., a higher indices) is actually correlated to a healthier environment [65].

Below, we use an ecological perspective to outline some of the key scientific and policy challenges that continue to hinder progress to link biodiversity and health through metrics, grouped by common themes:

A) Conceptual and Epistemological Barriers
  - socio-ecological connections are complex and multidimensional [68];
  - disciplines are divided into and communicate within separate silos (health science versus biodiversity science);
  - across these two communities there is generally limited knowledge on the governance history, theoretical foundations, and empirical evidence of the biodiversity-health nexus;
  - Indigenous metrics are not available or included in nature-health approaches;
B) Scientific and Methodological Challenges
  - it is difficult to quantify biodiversity with one single ecological indicator capturing all the dimensions of biodiversity [14], and therefore comprehensively account for ecosystem integrity
  - a substantial number (over 570) of biodiversity metrics exist and agreement on how to practically utilize these metrics, even for conservation purposes, has not been reached [69];
  - what kind and how much of biodiversity loss will reduce ecological resilience may vary across systems and regions of the world [70];
  - it is difficult to quantify all health outcomes, especially chronic and cumulative diseases, and also the spectrum of mental, emotional, spiritual and developmental aspects (such as inspiration and learning), or the diverse values of nature for human wellbeing [25,26,71];
  - the importance of a holistic connection between biodiversity and ecosystem function is often left out of market-based biodiversity valuation [26,72–74]; ‘nature’s contributions to people’ which is a concept that parcels out 18 contributions [19] and elements of natural capital, commoditize isolated benefits without attributing them to ecosystem integrity;
C) Data, Infrastructure, and Capacity Issues
  - national health data may be limited to reportable diseases, which typically leave out non-communicable and chronic diseases like inflammatory disorders, renal, endocrine, neurological, or mental health disease, and focus largely on communicable water-, food-, or vector-borne and transmissible disease;
  - data may be unavailable, unreliable, and require processing, or may be limited by patient confidentiality [58];
  - assessment of the relationship between health and nature includes the potential, character, and quality of positive and negative exposure as well as its duration, which cannot always be estimated and are not measures well adapted to perform in study settings;
  - decision makers need simple metrics which may lead to challenges balancing scientific rigor and policy applicability in metric choice;
  - funders may be interested in only one aspect of health or one element of biodiversity, yielding tools that zoom in on biodiversity, rather than explaining or advancing a holistic perspective of the value of ecosystems and ecosystem functions and services;
D) Institutional, Political and Educational Barriers
  - territorialism and siloed mandates in policy domains leads to avoidance of responsibility when it comes to integrated concepts and capacity building, such as biodiversity and health;
  - it is not feasible to have a global apex target for biodiversity such as there is for climate change goals (i.e., CO2 emission target or temperature target);
  - national governments have not adopted a clear mandate for integrated biodiversity-health indicators;
  - noncommunicable disease is substantially proportionally higher at national and global levels but policy investment tends to focus on communicable disease [75,76];
  - the communicable disease field places greater attention on vector-borne (e.g., mosquitos and zoonoses) rather than non-vector borne infectious disease (e.g., pathogens carried by floods) while both can be linked to biodiversity and ecosystem management [77].

Recognizing, and where possible, addressing these challenges is essential to develop metrics that are not only scientifically sound but feasible across policy contexts. Without targeted efforts to overcome these barriers, integrated biodiversity–health metrics will remain conceptually important but practically out of reach.

### Integrated metrics: Scientific basis

Effective biodiversity-health metrics must be theoretically grounded and capture biodiversity at multiple levels. This section evaluates ecological theories that can be considered in the development of integrated science-based indicators on biodiversity and health.

Several existing hypotheses highlight the positive role of biodiversity on human health at the local scale; the *Insurance Hypothesis* [78] suggests that biodiversity supports the resilience of ecosystems, helping with the provision of essential ecosystem services that directly affect human health; the *Biodiversity Hypothesis* [79] proposes that being exposed to a diversity of microorganisms, particularly during childhood, is suggested to improve immune function; the *Dilution Effect Hypothesis* [80–82] proposes that higher levels of biodiversity can reduce the transmission of infectious diseases by reducing the densities of vectors and hosts. In contrast, the *Harm Hypothesis* [23], predicts that biodiversity can have negative impacts on human health. For example, regions with elevated levels of biodiversity may host a larger number of animal vectors transmitting diseases, including for example Ebola, dengue, leishmaniosis or Lyme disease among others, although often, relationships between biodiversity measures and disease prevalence are context-dependent [83,84]. The *Buffering Hypothesis* describes the features and interactions in the natural world that physically buffer adverse effects to humans—for instance, the health benefits ecosystems provide by mitigating air or water pollution or urban heat [12]. The *Developmental Origins of Disease Hypothesis* suggests that environmental exposures, especially during early development, can interact with genetic factors and influence long-term health outcomes, implying that a degraded ecosystem can be health-determining across the life course [85]. However, all those hypotheses we have described are mainly supported by correlational evidence. Experimental based approaches, longitudinal studies and underlying mechanisms that link biodiversity to health are needed to explore their support and applicability.

Together, these hypotheses illustrate why the development of biodiversity–health indicators must begin with a clear theoretical foundation, ensuring that metrics are grounded in the mechanisms that effectively shape health outcomes across ecological context and national circumstances.

### Integrated metrics: Principles, characteristics, and policy relevance

Metrics should be fit for purpose. This section outlines principles to guide the development or selection of integrated biodiversity–health metrics. Clarifying these characteristics can help researchers, practitioners, and governments make better choices in monitoring the biodiversity–health nexus, designing more effective interventions, and supporting implementation in diverse policy contexts and NBSAPs.

Core principles for biodiversity–health metrics:

- Theoretically grounded: Metrics should be based on ecological and health hypotheses with empirical support (e.g., Insurance, Dilution, Biodiversity, Buffering, or Harm Hypotheses etc).
- Reflect multiple levels of biodiversity: They should capture biodiversity at genetic, species, community, and ecosystem levels—not only species richness.
- Link biodiversity to health outcomes, ideally through evidenced exposure-response models: To indicate how changes in biodiversity affect risks or benefits to human health (e.g., chemical exposure, microbial exposure, vector abundance, pollination, food security, etc) expressed in health measures (e.g., disability-adjusted life years (DALYs), quality-adjusted life year (QALYs), or years of life lost (YLLs)).
- Policy relevant: To inform decision-makers by correlating with real-world health risks or benefits (e.g., waterborne disease risk, nutritional impacts of pollinator loss).
- Scalable and context-sensitive: Metrics should be usable across different spatial scales (local to global) and adaptable to national or regional contexts.
- Consistent, repeatable, and comparable: They must provide reliable results across time and geography, enabling long-term monitoring and cross-country comparisons.
- Aligned with existing frameworks: Where possible, metrics should build on or align with global indicator frameworks (e.g., IPBES, Essential Biodiversity Variables, SDGs, WHO Global Health Observatory) to reduce duplication and facilitate uptake.
- Feasible and actionable: Metrics should be practical for use in resource-limited settings and clearly guide policy or management decisions.

### Analyzing holistic approaches to monitoring biodiversity and health integration in policy and implementation

**Fig 2:**
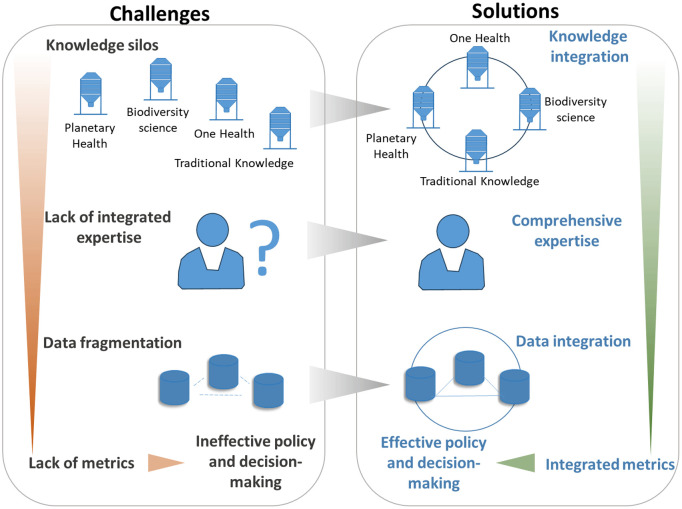
Challenges and Solutions for Biodiversity and Health Policy Integration.

Several communities supporting interconnection between society and the environment are working from distinct perspectives on the nature-health nexus and this is apparent in the difference in metrics they use. Here we describe science-policy metrics by conservationists, Indigenous scholars, One Health leaders, and by planetary health experts as a way to enhance awareness of parallel efforts within and between communities. Convergence and collaboration of technical work on integrated metrics across these communities will strengthen the field. On the other hand, continued separation of these communities or competition between them negatively influences the development of the field, limiting cohesive risk and cost management that governments need to optimize public health. See Fig 2.

We frame the approaches of these four communities by grey literature of the United Nations (UN). UN grey literature is a gateway to recognition in international environmental law and therefore reflects science-policy authority. The material used is not exhaustive but is indicative of the current narratives existing in multilateral dialogues across environment and health forums. Each section includes light analysis of opportunities and gaps of each approach to contribute to biodiversity and health indicators under the CBD.

### Global biodiversity governance indicators

This section analyzes the evolution of integrated biodiversity-health indicators under the CBD. To our knowledge, there is no previous analysis of this type in biodiversity or health literature. This information provides an important foundation for advancing future work on health under the CBD. In February 2025 the CBD Secretariat put out a call for input on integrated health indicators underscoring the need for a baseline for collaboration [86].

The biodiversity governance community centers on the CBD, a global forum, and the implementation of NBSAPs, both of whose agendas are steered by 196 government parties. Stakeholders to the CBD traditionally include conservationists, environmental managers, protected areas and species specialists, and a range of natural scientists, such as ecologists, marine biologists, soil conservationists, and many others. The conservation community has long identified connections to health, however, the development and evolution of health indicators under the CBD has been limited. It has also been historically overlooked by the public and global health communities, including by One Health and planetary health experts.

While health elements were included in many biodiversity decisions since the CBD entered into force in 1994 [39], intergovernmental agreement to develop biodiversity and health indicators was first reached in 2012 [2,87]. This was in association with implementation of the 2010 Aichi Biodiversity Targets, which was the first global blueprint to address and mitigate biodiversity loss worldwide [87]. The “flexible framework” adopted as guidance for the implementation of the Aichi Targets steered governments to develop their own national biodiversity targets [88]. But it also resulted in an orientation of national commitments on biodiversity that was variable worldwide [89]. Follow-up analyses in 2018 and 2019 showed that the health component of NBSAPs was weakly represented [89–92]. Lamentably, while one ambition of the 11th conference of the parties to the CBD (COP 11) in 2012 was to stimulate collaboration on integrated health work with a range of institutions [2], dialogue on health-related indicators continued without committed action through subsequent CBD COPs for over a decade [2–6,8]. See S1 Table.

The second global blueprint to address biodiversity loss, the Kunming-Montreal Global Biodiversity Framework (GBF) was adopted in 2022, stipulating that health should be mainstreamed across all its goals and targets [1]. This means health does not have a stand-alone goal, target, nor therefore its own monitoring process [90,93]. Nevertheless, intergovernmental negotiations achieved a few health-related qualitative and quantitative indicators within the GBF Monitoring Framework, among dozens of compulsory headline and voluntary supporting indicators [93–95].

Table 1 shows the evolution of health and health-related indicators under the CBD between 2010 and 2024. CBD health indicators in 2012 centered on five elements for *medicine* (species), *water* (freshwater ecosystem state and calculation of drinking water), and *food* (species consumed and utilized for pollination, and calories obtained) [87]. The 2025 GBF Monitoring Framework includes a mix of ten health-related elements (three compulsory and seven voluntary) on: *nature’s contributions to people* (including health-related ecosystem services); *food* (proportion of fishstocks sustainable, number of plant and animal genetic resources for food and agriculture conserved, availability of pollinator species); *water* (proportion of water bodies with good water quality, water stress, and water safety and associated mortality); *human-wildlife conflict*; *pollution* (pesticide concentration/aggregate toxicity, particulate air pollution); and *disaster mortality* [93].

**Table 1:** Evolution of health indicators under the Convention on Biological Diversity 2010-2024: Aichi Biodiversity Targets to the Global Biodiversity Framework.

*National Indicators*			
**AICHI BIODIVERSITY TARGETS** [87] (2010)			**KUNMING-MONTREAL GLOBAL BIODIVERSITY FRAMEWORK** [1] (2022)
Flexible Guidance for NBSAPs [88]			**Adopted Monitoring Framework for updating NBSAPs **[93]
**Trends in extinction risk and populations of species that provide essential services**		• species used for food and medicine, pollination	**Compulsory ** *direct health-related indicators* • number of countries monitoring the maintenance and enhancement of nature’s contributions to people • proportion of fish stocks at sustainable levels • pesticide environment concentration/ aggregated total applied toxicity
**Trends in benefits from ecosystem services**		• Wellbeing indicator for the environment • % of water bodies with good ambient water quality	**Voluntary ** *direct health-related indicators* • proportion of bodies of water with good ambient water quality and • levels of water stress and safely managed drinking water and associated mortality rate • human-wildlife conflict • annual levels of particulate air pollution • deaths due to disasters *indirect health-related indicators* • number of plant and animal genetic resources for food and agriculture secured in conservation facilities • pollinating species availability
**Trends in the degree to which ecosystem services provide for the needs of women, Indigenous and local communities, and the poor and vulnerable**		• inadequate access to food – average dietary energy supply adequacy • % of population using safely managed drinking water services	

*Source: authors. Bold categories are as listed in the CBD documents. “Health-related” measures were identified by the authors from the Monitoring Framework as those directly relating to health ecosystem services, to food or water availability, or to mortality and morbidity. These measures were not identified as “health-related” in the Monitoring Framework. The indicators listed in the right-hand column as “indirect” (genetic resources for food and agriculture, pollinators) underpin food supply and therefore human health; however an additional calculation is required to translate these ecosystem-level indicators into food system impact and then into the number of people affected and/or health outcome and therefore is determined by the authors to be an “indirect” measure.*

While the GBF tracks more health-related items than those associated with the Aichi Targets, there does not seem to be continuity on health between the two blueprints. The metrics in the GBF Monitoring Framework also fall short of formally recognizing public health domains, environmental determinants of health, or environmental burden of disease. They do not comprehensively reflect the literature base on biodiversity and health and key science resources available during the development of the GBF Monitoring Framework. For instance, these resources include the World Health Organization (WHO) and CBD state of knowledge review commissioned by CBD parties [3,17]; material from IPBES’ Global Assessment including a dedicated chapter on nature’s contributions to people [19,96]; the IPBES Assessment of Diverse Values and Valuation of Nature [26,74]; the scope of health considerations in the definition of nature’s contributions to people [94]; or the many published conceptual frameworks on environment and health [12–37].

The adoption of the CBD Global Action Plan on Biodiversity and Health in 2024 provides a new opportunity to reinforce comprehensive tracking on biodiversity and health interlinkages [38,97]. The Plan was anticipated to be negotiated prior to the adoption of the GBF, but largely due to the COVID-19 pandemic was postponed until afterwards. While adoption of the Plan prior to the GBF might have led to a stronger health positioning in the GBF now the independence of the Plan creates a parallel opportunity to align and deepen country efforts to scope and integrate health into NBSAPs.

Ongoing development of supporting indicators under the GBF Monitoring Framework and new work to identify indicators to track the Global Action Plan on Biodiversity and Health should be cohesive and focus on filling substantive gaps. New information, such as the unnegotiated CBD Secretariat “Monitoring Elements” (See Annex II) [7] and the IPBES Thematic Assessment Report on Interlinkages Among Biodiversity, Water, Food, and Health and on Transformative Change [71,98], should also be considered. Going forward, indicators will be used in the development of updated NBSAPs, in national reporting on the indicators to the CBD, and the CBD’s global review of indicator reporting [99,100]. Institutional knowledge and synchronicity of policy processes across the CBD and IPBES will continue to be important.

### Progress tools of Indigenous Peoples

Although traditional public health scholarship overlooks interconnectedness, a growing body of Indigenous scholarship puts a spotlight on it. Metrics relating Indigenous human health and values to the environment have been introduced under the UN Permanent Forum on Indigenous Issues (UNPFII) [30,101].

Various frameworks from Indigenous scholars point to potential qualitative measures for progress on Indigenous determinants of planetary health [25] and Indigenous determinants of health [30]. The “Implementation Tool on Indigenous Determinants of Health”, a set of qualitative, institutional and governance indicators, was recognized under the Indigenous advisory body to the UN Economic and Social Council [101]. It contains quantifiable social elements but not science-based metrics. See Box 2 which aggregates several recommendations on Indigenous and traditional knowledge and knowledge holders related to the health-environment nexus.

Interconnection between health, environment, and wellbeing is inherent to Indigenous worldviews, worldwide [25,102–104] but Indigenous science-based metrics are underdeveloped. The challenge is intrinsic. While reciprocity is one way the relationship between humans and nature is defined by Indigenous knowledge holders, metrics are unable to describe this interconnection, as intrinsic value, and “oneness” is unquantifiable; attempts at its measurement may even undermine its understanding. This is an area of scholarship needing greater attention and broader application in multilateral agreements on health and environment.

**Box 2: Measuring Interconnection and ‘Oneness’: Recommendations on incorporating Indigenous perspectives on the health-environment nexus into policies, plans, and strategies [101,105–107]**

**Table:** 

• recognize human interconnectedness with Nature (i.e., human health is completely and utterly dependent on a healthy planet); • recognize Indigenous Natural Law (i.e., “a comprehensive ethical framework that defines the codes of conduct necessary for maintaining a peaceful, thriving, and co-operative society grounded in love and reciprocity”); • incorporate values on nature and culture into evaluation plans to develop effective, culturally relevant health programmes; • incorporate Indigenous-specific health and wellness measures and indicators, which include interconnection with the environment, in data-collection and tracking; • include spirituality, culture and language in health research; • recognize and acknowledge the interconnectedness of mental, physical, spiritual, and environmental factors; • acknowledge that holistic health factors affect multiple generations (i.e., consideration of intergenerational impacts); • recognize the role of Indigenous elders and children in the intergenerational transmission of traditional ecological knowledges;	• consider the health of Indigenous Peoples as a proxy for environmental stewardship, given their role in stewarding biodiversity and the remaining old-growth forests; • track trends in land-use change and land tenure in the traditional territories of Indigenous and local communities [78]; • recognize and support Indigenous languages, “the blueprint of traditional ecological knowledges” [101]; utilize language trends (diversity and numbers) as an indicator for traditional knowledge and as a proxy for biodiversity loss [105,106]; • track trends in the degree to which traditional knowledge and practices are respected through their full integration, safeguards and the full and effective participation of Indigenous and local communities in national implementation [107], including trends in the practice of traditional occupations; • respect the feminine (i.e., women and other female identifying genders as key knowledge holders with respect to ecology and traditional medicine).

### National One Health indicators

One Health has received significant investment over the last two decades since the origin of the concept, followed by heightened attention in recent years due to the COVID-19 pandemic and the expansion of the definition to consider the environment in 2022. While numerous studies evaluate aspects of One Health, holistic evaluation methods and indicators that encompass the “interconnection” of global health and environmental challenges remain less developed. For instance, in a review of more than 1800 papers Baum et al (2017) found that standardized approaches to evaluating One Health were lacking [108]. Pandemic prevention and reducing risks of emerging infectious disease remain a focus of One Health assessment. This is seen in cost-benefit analysis [109] as well as in explanations of the underlying One Health rationale for public health tools, even those that incorporate a range of environmental factors, such as the Global One Health Index [110].

Recently, new metrics and implementation tools for One Health were put forth by an independent UN advisory group, the One Health High-Level Panel of Experts (OHHLEP). OHHLEP advises the cohort of agencies who together address the health-environment nexus as the Quadripartite (WHO, UN Environment Programme, Food and Agriculture Organization of the UN, World Organization for Animal Health) and is unassociated to a specific forum or multilateral agreement. In 2023 the Panel released a guidance document on implementing the One Health approach at the national level [111], which includes a template for a national One Health workplan [112]. The guidance document and template recognize three key indicator areas in the OHHLEP theory of change [28,111,112]:

1. qualitative indicators describing the state and development of policy architecture to support One Health;
2. establishment or enhancement of national-level integrated One Health information and early warning systems on diseases, antimicrobial resistance, and other health threats;
3. quantitative tracking at the national level for *disease* (incidence, emergence, prevalence and spread); *wildlife and environment monitoring*; *drivers of health threats* (e.g., biodiversity loss, soil and water degradation, water scarcity, land use change and habitat conversion, pollution and climate change); *data on food production* (systems, trade); and *social factors* (migration, knowledge, attitudes, practices, political and socioeconomic determinants of health).

OHHLEP promotes robust development of qualitative progress measurement indicators for national One Health policy architecture but does not prescribe or advocate specific integrated science-based indicators. See Table 2 for a condensed view of the OHHLEP indicators.

**Table 2:** Framing for indicator development under the OHHLEP template for a national one health workplan [28,111,112].

*OHHLEP Joint Plan of Action*						
Tracks	Enhancing One Health capacities to strengthen *health systems*	Reducing the risks from emerging and re-emerging *zoonotic epidemics* and *pandemics*	Controlling and eliminating endemic zoonotic, neglected *tropical and vector- borne diseases*	Strengthening the assessment, management and communication of *food safety* risks	Curbing the silent pandemic of *antimicrobial resistance*	Integrating the *environment* into One Health
**Theme**	*Qualitative Indicators Suggested by OHHLEP for Each Action Track at the National Level*					
governance	• guidance and tools for establishing multisectoral One Health coordination mechanisms for collaborative governance					
policies & legislation	• science–policy interfaces to ensure that scientific evidence is translated into policies and legal frameworks					
	• gap analyses of policies, legislation, financial resources and operations relevant for each sector					
	• participate in standard-setting related to One Health					
financing	• financial needs to address research gaps and priorities, develop a research agenda, and build One Health capacity					
advocacy	• awareness raised among key stakeholders about risks and benefits of the One Health approach					
organizational & institutional development	• leverage, use and results of existing capacity evaluation tools and roadmaps					
implementation	• coordinate One Health efforts at global, regional and national level					
sectoral integration	• link and integrate One Health sectors, tools, existing national action plans, and existing technical programmes					
data & evidence	• standard operating procedures, operational tools and resources for harmonized One Health research and data collection					
information systems	• standard operating procedures, operational tools and resources to conduct and strengthen targeted One Health integrated multisectoral surveillance and mapping					
knowledge exchange	• effective communication structures and information and data-sharing systems					

*Source: [112]; The information from the source was adapted and truncated for the focus of this article

The One Health Joint Plan of Action 2022–2026 [28] points to aligning indicators with any existing monitoring and evaluation frameworks as well as with indicators for the SDGs. This is critical for resource limited settings. An ongoing limitation of OHHLEP’s advisory is the predominant focus on communicable disease links to ecosystems rather than noncommunicable disease links, and also the incremental progress on its sole workstream on the environment. There is a conceptual difficulty with the definition of One Health (“interconnection between the health of humans, animals, plants and the environment”) and the implementation plan to “integrate the environment into One Health”, inferring the environment is not central, as defined. Lamentably, funding consistently backs the inclusion of environment merely as a risk factor for the other five priority areas related to infectious disease and this further limits the application of One Health as an “interconnection” approach. Ultimately, this also means One Health predominantly focuses on health as opposed to environment or environmental change and planetary crises [49]. It will be difficult for integrated ecosystem-health indicators under OHHLEP to encompass the realities of environmental burden of disease and diverse values of biodiversity until its scope includes a stronger consideration and balance of natural ecosystems and environmental drivers. This needs greater attention.

### Planetary health measurement tools

The dialogue on planetary health and the need for the human-environment nexus to become a focus in sustainable development in the UN System is gaining increasing attention [113]. But policy tools for planetary health are not as developed as integrated metrics for biodiversity, Indigenous Peoples, or for One Health and have not yet been formally introduced or proposed within global environmental governance or global health monitoring bodies. At the same time, the Intergovernmental Panel on Climate Change (IPCC) has defined and recognized the planetary health term meaning governments have agreed to its concept and could seek to build indicators accordingly [45,114].

A transdisciplinary workshop held in 2019 convened 59 experts to assess the state of knowledge and development of the ‘Planetary Heath Watch’ concept and integrated monitoring of the “health effects of, and responses to, global environmental changes” [58]. More than 150 monitoring initiatives were reviewed ahead of the workshop but little integration of data between environmental change and human health exposures or outcomes was found and no explicit indicators to link biodiversity change and human health were identified.

Planetary Health Rapid Impact Assessments (PHRIA) are one tool that could facilitate government understanding of interventions that might contribute to healthy ecosystems, based on elements of both health and natural systems. The PHRIA are two coupled tools using a planetary health framework comprised of three natural planetary boundaries, three determinants of human wellbeing, and both the power and governance of implementation [115]. The first tool generally informs governments whether an action is degenerative or regenerative to public health and planetary health. The second tool describes the probability and magnitude of potential impact of a policy intervention. Brousselle et al. [115] argue that the utilization of both tools together can be used to create a “Planetary Health Index” for policy actions. While the tools do not include all planetary boundaries [116], nor all dimensions of health determinants, they are flexible and may allow a government to prioritize a few key elements or indicators of public health, ecosystem management, and planetary health. They may also be comparative, to consider tradeoffs.

An increasingly common metric tool utilized by planetary health scholars and the WHO at global and regional scales is the environmental burden of disease. This metric captures the planetary health “state of natural systems” upon which human health depends. An initial calculation published by WHO found the contribution of environmental degradation to the global burden of disease to be approximately 25 percent, with an increase to 28 percent when only children were considered [117]. Burden of disease has been reported by UN regional offices ranging from 13 to 28 per cent [118].

These calculations are generally understood to be underestimates, limited by: the availability of data (for example, on chemical exposures); unaccounted for health conditions (such as sub-lethal neurological or endocrine disease); poorly understood pathophysiology of cumulative negative exposures to environmental degradation of similar or varied types; and the scope of environmental factors included in the calculation. Fleming et al. (eds) (2023) describe the Global Burden of Disease study as an influential, worldwide, accessible resource and that its “coverage of environmental risks is still relatively sparse” [31].

A national assessment of environmental burden of disease was first proposed as a progress measure for the CBD’s Global Action Plan on Biodiversity and Health in its initial round of negotiations in 2022 and again offered to negotiators for consideration in 2024 [118,119]. Including this metric in NBSAPs would mandate collaboration of environment and health experts and government offices in a new way. It has promising inroads across policy arenas. The metric has traction within the marine science community where it is used to assess the role of the seas, coasts, and ocean as a determinant of health [31]. The metric is also already advancing under the UN Framework Convention on Climate Change (UNFCCC) where parties planted the climate burden of disease as a fundamental aspect of the Global Goal on Adaptation [120]. One 2030 target of the Goal stipulates parties to significantly reduce climate-related morbidity and mortality, particularly in the most vulnerable communities [120]. The Global Framework for Chemicals also leans on the environmental burden of disease (“attributable to chemicals and waste”) as a headline indicator, though underlying, supporting indicators and targets have not yet been identified [121]. The Sendai Framework for Disaster Risk Reduction also uses a mortality metric related to natural hazards [122].

Much more investment is needed to make planetary health assessments and environmental burden of disease calculations complete. Moreover, planetary health advocacy is limited in intergovernmental dialogues and negotiations where it potentially could be incorporated into multilateral decisions.

### Looking ahead

At the global level, nearly 200 governments have called for integrated policy and implementation on biodiversity and health for more than a decade. This work has been unable to move forward without development and commitment to indicators and this gap is an oversight.

Regardless of whether intergovernmental bodies commit to an integrated package of indicators, metrics, and progress measurement tools on ecosystems and public health (such as under the CBD), national government officials will continue to make decisions on the environment, many of which will be health determining. Equipping a government on whether and how to choose environmental impacts, some of which can and will be irreversible, while keeping in mind the health and wellbeing of their populations, is important. These metrics can also inform governments of vulnerability, risks, and threats to their populations from increasing ecological changes. Moreover, they can inform all stakeholders on pathways to optimize the well-being, growth, and development of children and youth.

Various scholars identify priority areas at the nature-health nexus that could inform indicator development. Nishi, Subramanian, and Gupta (2022) suggest that focusing on locally-integrated solutions is a way to best reflect the dynamic interdependencies of ecosystem components and human uses across spatial scales and sectors [68]. This may also maximise synergies and minimize tradeoffs for the multidimensional aspects of human well-being [68]. Alternatively, Lajaunie and Morand (2021) suggest framing solutions around planetary limits and environmental disparities and justice [48]. Gittleman (2024) considers the need to center on overlapping hotspot issues by unifying global surveillance and database development [49]. Eberle et al. (2025) orient around human assumptions and social structures and the inner and outer levers needed for deep durable change in both [123].

We propose a few near-term priorities to effectively address the science-policy challenges ahead and maximize opportunities to enhance interdisciplinary dialogue and integrated governance at any scale:

- Calls for integrated biodiversity and health metrics from nearly 200 governments under the CBD for more than a decade need to be taken seriously. Metrics are needed for monitoring the Global Action Plan on Biodiversity and Health and for tracking and reporting on the Kunming-Montreal Global Biodiversity Framework. Allowing NBSAPs to finalize without consideration of biodiversity-health indicators, or comprehensive ones, is a missed opportunity that will impact global, regional, and national approaches to planning, investment, and stewardship of nature for more than a decade. Intersessional preparations of the CBD is a critical time for informing governments, the CBD Secretariat, and the negotiating process itself on comprehensive indicators, metrics, tools, and scientific developments.
- Funding and increased investment is needed from donors, governments, and the private sector to develop and share best practices on integrated ecosystem and health monitoring elements. Support is needed to build capacity among social and natural scientists to effectively work together and to create, update, and report on integrated qualitative and quantitative measures [97]. Major funders of biodiversity conservation and restoration and interested governments should fund aligned measurement and reporting on health outcomes. Likewise public health should broadly consider biodiversity-health interlinkages, including the impacts of health systems, and health interventions for climate action, on biodiversity.
- Robust integrated indicators must be accompanied by a standardized framework that ensures consistency, reliability, and relevance across different regions and contexts. This involves: interdisciplinary collaborations to foster cooperation among ecologists, health professionals, and policymakers; the collection and integration of data across disciplines, ensuring that biodiversity and health data are harmonized; designing metrics that are flexible and can be adjusted as new data and insights become available; and ensuring that metrics are actionable and aligned with policy needs, providing clear guidance for decision-makers on how to integrate biodiversity and health considerations effectively. Academic institutions can convene disciplines of biodiversity, One Health, planetary health, and Indigenous and traditional knowledge scholars [101,124] in a balanced format with related government departments and municipal or national level authorities to mobilize interdisciplinary dialogue;
- Countries must commit under multilateral bodies to utilize three types of monitoring elements on biodiversity and public health to ensure greatest flexibility and comprehensiveness: qualitative indicators, quantitative indicators, and integrated science-based metrics and progress measurement tools. An overall goal, such as national environmental burden of disease would be transformative.

Biodiversity and health metrics are needed across the multilateral environmental system. Integrated biodiversity and health metrics are needed to inform the UNFCCC Global Goal on Adaptation (United Arab Emirates Framework for Global Climate Resilience) [120], non-economic loss and damage assessments [125,126], and national disaster risk reduction plans [127]. Assessing scenarios of ecosystem change is a foundational activity for fostering climate-resilient communities [114]. Changes in nature are indicators of broader climatic change, meaning there is urgency to understand how biodiversity and health metrics can be measured, communicated, and strengthened. These metrics will help drive demand and application of ecosystem-based and nature-based solutions to mitigate and adapt to climate change and achieve the human right to a healthy environment. Integrated biodiversity and health metrics are also needed to monitor the unsound management of chemicals and waste as laid out in the intergovernmental goals of the Global Framework on Chemicals [121]. The metrics can be applied to realizing convergence between goals on chemicals and waste and on biodiversity [128]. Another example, is that integrated biodiversity and health metrics and progress measurement tools should be used efficiently to inform the commitments and planning of environmental ministries and of health ministries who will aim to fulfill strategic objectives under the WHO 14^th^ General Programme of Work to address social, economic, environmental and commercial determinants of health and the root causes of ill health in key policies across sectors [129].

In addition to supporting individual multilateral agreements, integrated metrics may be a concrete way to bring different frameworks together and identify synergies and efficiencies in multilateral environmental agreements. This is a priority raised at the highest levels of environmental governance in the UN system [130] and in a recommendation adopted by governments on the monitoring framework for the Global Biodiversity Framework [95]. Ultimately, currently separate health-environment communities of practice within biodiversity conservation, Indigenous scholarship, One Health, and planetary health should strive to recognize gaps in their independent approaches and identify opportunities for cohesive, synergistic, and balanced collaboration as one holistic and co-dependent community of practice. Metrics are a good starting point for collaboration. We caution the development and/or application of siloed metrics that are certain to result in imbalanced perspectives of the multiple ecosystem service needs and values of people and imbalance in comparative values of quantity versus quality. Isolated approaches are also likely to overlook unequal access among nature’s beneficiaries as well as the rich diversity of local contexts [68].

As we near the post-2030 sustainable development period clear foundations for integrated measurement must be rooted in national policy architectures to demonstrate a path toward coordinated environment and health decision making. The risks of temperature extremes, biodiversity loss and ecosystem collapse, and natural resource shortages are expected to be top threats to our global society and local communities in the next decade [54]. Understanding and applying health indicators, metrics, and measures to global environmental changes is critical to our resilience.

## Supporting information

S1 Table Intergovernmental Requests for Biodiversity-Health Indicators Under the  Convention on Biological Diversity Between 2012 and 2024. (DOCX)

## References

[1] CBD. Decision 15/4: Kunming-Montreal Global Biodiversity Framework, 15^th^ Conference of the Parties Part II, 7-19 December, Montreal, Canada; 2022. https://www.cbd.int/doc/decisions/cop-15/cop-15-dec-04-en.pdf

[2] CBD. Decision XI/6: Cooperation with other conventions, international organizations, and initiatives, 11th Conference of the Parties, 8-19 October, Hyderabad, India; 2012. https://www.cbd.int/decision/cop/default.shtml?id=13167.

[3] CBD. Decision XII/21: Biodiversity and human health, 12th Conference of the Parties, 6-17 October, Pyeongchang, Republic of Korea; 2014. https://www.cbd.int/doc/decisions/cop-12/cop-12-dec-21-en.pdf

[4] CBD. Decision XIII/6: Biodiversity and human health, 13th Conference of the Parties, 4-17 December, Cancun, Mexico; 2016. https://www.cbd.int/decisions/cop/13/06/01.

[5] CBD. Decision 14/4: Biodiversity and human health, 14th Conference of the Parties, 17-29 November, Sharm-El-Sheikh, Egypt; 2018. https://www.cbd.int/decision/cop/default.shtml?id=13643

[6] CBD. Decision 15/29: Biodiversity and human health, 15th Conference of the Parties Part II, 7–19 December, Montreal, Canada; 2022. https://www.cbd.int/decisions/cop?m=cop-15

[7] CBD. Biodiversity and Health (CBD/SBSTTA/REC/26/9), Recommendation to the 16th Conference of the Parties, 26th Meeting of the Subsidiary Body on Scientific, Technical, Technological Advice, 13–18 May, Nairobi, Kenya; 2024 https://www.cbd.int/doc/recommendations/sbstta-26/sbstta-26-rec-09-en.pdf.

[8] CBD. Decision 16/19 Biodiversity and Health, 16th Conference of the Parties to the Convention on Biological Diversity, Calí, Colombia; 2024. https://www.cbd.int/health/GAP.shtml

[9] Fairbrass A, Mace G, Ekins P, Milligan B. The natural capital indicator framework (NCIF) for improved national natural capital reporting. Ecosystem Services. 2020;46:101198. doi: 10.1016/j.ecoser.2020.101198

[10] Dasgupta P. The Economics of Biodiversity: The Dasgupta Review – Full Report. London, UK, UK Treasury; 2021. https://www.gov.uk/government/publications/final-report-the-economics-of-biodiversity-the-dasgupta-review

[11] Al-Kibsi G, Casoli A, Crowhurst S, Jayaram K, Levy C, Pacthod D, et al. Representing views from McKinsey Sustainability. Climate Transition Impact Framework: Essential elements for an equitable and inclusive transition. Published by McKinsey Sustainability; 2023.

[12] Robinson JM, Breed AC, Camargo A, Redvers N, Breed MF. Biodiversity and human health: A scoping review and examples of underrepresented linkages. Environ Res. 2024;246:118115. doi: 10.1016/j.envres.2024.118115 38199470

[13] Wilson EO. Biophilia: the human bond with other species. Boston: Harvard University Press. 1984.

[14] Millennium Ecosystem Assessment. Ecosystems and Human Well-being: Biodiversity Synthesis. World Resources Institute, Washington, D.C., USA; 2005. https://www.millenniumassessment.org/en/index.html

[15] Millennium Ecosystem Assessment. Ecosystems and Human Well-being: Health Synthesis, Geneva, Switzerland: World Health Organization; 2005. https://www.millenniumassessment.org/en/index.html

[16] Whitmee S, Haines A, Beyrer C, Boltz F, Capon AG, de Souza Dias BF, et al. Safeguarding human health in the Anthropocene epoch: report of The Rockefeller Foundation-Lancet Commission on planetary health. Lancet. 2015;386(10007):1973–2028. doi: 10.1016/S0140-6736(15)60901-1 26188744

[17] WHO and CBD. Connecting Global Priorities: Biodiversity and Human Health, A State of Knowledge Review, World Health Organization, Geneva, Switzerland; 2015. https://www.cbd.int/health/SOK-biodiversity-en.pdf

[18] UNEP. Healthy Environment, Healthy People. Thematic report, Ministerial policy review session, 2nd Session of the United Nations Environment Assembly, 23–27 May, Nairobi, Kenya; 2016. https://wedocs.unep.org/bitstream/handle/20.500.11822/17602/K1602727%20INF%205%20Eng.pdf?sequence=1&amp%3BisAllowed=

[19] IPBES. Global assessment report on biodiversity and ecosystem services. E. S. Brondizio, E.S., Settele, J., Díaz, S., and Ngo, H.T. (eds). IPBES Secretariat, Bonn, Germany; 2019. doi: 10.5281/zenodo.3831673

[20] UN General Assembly. Human rights depend on a healthy biosphere, UN Special Rapporteur report on human rights and the environment Report on the Issue of Human Rights Obligations Relating to the Enjoyment of a Safe, Clean, Healthy and Sustainable Environment, 75th Session of the UN General Assembly (A/75/161), New York, NY, USA; 2020. https://documents.un.org/doc/undoc/gen/n20/184/48/pdf/n2018448.pdf

[21] UN General Assembly. Resolution 76/300, The human right to a clean, healthy and sustainable environment, 76th Session of the UN General Assembly, 28 July, New York, NY, USA; 2022. https://www.un.org/en/ga/76/resolutions.shtml

[22] UNICEF. The Climate Crisis is a Child Rights Crisis: Introducing the Children’s Climate Risk Index. New York, NY, USA; 2021. https://www.unicef.org/media/105376/file/UNICEF-climate-crisis-child-rights-crisis.pdf

[23] Marselle MR, Hartig T, Cox DTC, de Bell S, Knapp S, Lindley S, et al. Pathways linking biodiversity to human health: A conceptual framework. Environ Int. 2021;150:106420. doi: 10.1016/j.envint.2021.106420 33556912

[24] Keune H, Payyappallimana U, Morand S, Rüegg SR. One Health and Biodiversity. Transforming Biodiversity Governance. Cambridge University Press; 2022. p. 93–114. doi: 10.1017/9781108856348.006

[25] Redvers N, Celidwen Y, Schultz C, Horn O, Githaiga C, Vera M, et al. The determinants of planetary health: an Indigenous consensus perspective. Lancet Planet Health. 2022;6(2):e156–63. doi: 10.1016/S2542-5196(21)00354-5 35150624

[26] IPBES. Summary for Policymakers of the Methodological Assessment Report on the Diverse Values and Valuation of Nature of the Intergovernmental Science-Policy Platform on Biodiversity and Ecosystem Services. Pascual, U, Balvanera, P, Christie, M, Baptiste, B, González-Jiménez, D, Anderson, C, et al, editors. IPBES secretariat, Bonn, Germany; 2022. doi: 10.5281/zenodo.6522392

[27] IPCC. Health, Wellbeing, and the Changing Structure of Communities. In: Working Group II contribution to the Sixth Assessment Report of the Intergovernmental Panel on Climate Change. Climate Change 2022: Impacts, Adaptation and Vulnerability. Cambridge, UK; Cambridge University Press; 2022. pp. 1041–1170. https://www.ipcc.ch/report/ar6/wg2/chapter/chapter-7/

[28] FAO, UNEP, WHO, and WOAH. One Health Joint Plan of Action (2022-2026): Working together for the health of humans, animals, plants, and the environment. Rome, Italy; 2022. https://www.unep.org/resources/publication/one-health-joint-plan-action-2022-2026

[29] Liu M, Wei H, Dong X, Wang X-C, Zhao B, Zhang Y. Integrating Land Use, Ecosystem Service, and Human Well-Being: A Systematic Review. Sustainability. 2022;14(11):6926. doi: 10.3390/su14116926

[30] UNPFII. Note by the Secretariat: Indigenous determinants of health in the 2030 Agenda for Sustainable Development (E/C.19/2023/5), 22nd Session of the UN Permanent Forum on Indigenous Issues, 17-28 April, New York, NY, USA; 2023. https://undocs.org/Home/Mobile?FinalSymbol=E%2FC.19%2F2023%2F5&Language=E&DeviceType=Desktop&LangRequested=False

[31] Fleming LE, Alcantara Creencia LB, Gerwick WH, Ching Goh H, Gribble MO, Maycock B. Oceans and human health: opportunities and impacts. 2nd ed. Amsterdam, Netherlands: Elsevier Inc. 2023.

[32] United Nations Human Rights Council. Report of the Special Rapporteur on the implications for human rights of the environmentally sound management and disposal of hazardous substances and wastes (A/HRC/33/41), New York, NY, USA; 2016. https://undocs.org/en/A/HRC/33/41

[33] United Nations Human Rights Council. Analytical study on the relationship between climate and the full and effective enjoyment of the rights of the child, a Report of the Office of the High Commissioner for Human Rights, 35th Session of the Human Rights Council (A/HRC/35/13), 6-23 June, New York, NY, USA; 2017. https://ap.ohchr.org/documents/dpage_e.aspx?si=A/HRC/35/13

[34] United Nations Human Rights Council. Report of the Special Rapporteur on the issue of human rights obligations relating to the enjoyment of a safe, clean, healthy and sustainable environment, 37th session of the Human Rights Council (A/HRC/37/58), 26 February–23 March, New York, NY, USA; 2018. https://undocs.org/A/HRC/37/58

[35] United Nations Human Rights Council. Human rights depend on a healthy biosphere, Executive Summary, Special Rapporteur on human rights and the environment, Materials on the substantive elements of the right to a healthy environment based on thematic reports to Human Rights Council and the General Assembly (A/75/161—Executive summary), New York, NY, USA; 2020. https://www.ohchr.org/sites/default/files/2022-02/BiosphereSummary.pdf

[36] CBD. Supplementary information for the global action plan on biodiversity and health (SBSTTA/26/INF/3), 26th Meeting of the Subsidiary Body on Scientific, Technical, and Technological Advice, 13–18 May, Nairobi, Kenya; 2024. https://www.cbd.int/doc/c/5ce9/2fad/42ebbc40e73b0fd1b9738646/sbstta-26-inf-03-en.pdf

[37] Reis S, Morris G, Fleming LE, Beck S, Taylor T, White M, et al. Integrating health and environmental impact analysis. Public Health. 2015;129(10):1383–9. doi: 10.1016/j.puhe.2013.07.006 24099716

[38] CBD. Global Biodiversity Outlook 5. Montreal, Canada; 2020. https://www.cbd.int/gbo5

[39] Willetts E, Grant L, Bansard J, Kohler PM, Rosen T, Bettelli P, et al. Health in the global environmental agenda: A policy guide. International Institute for Sustainable Development; 2022. https://www.iisd.org/publications/health-global-environment-agenda-policy-guide

[40] CBD. Article 2: Use of Terms. Montréal, Canada; 1992. https://www.cbd.int/convention/text

[41] Hansen AJ, Noble BP, Veneros J, East A, Goetz SJ, Supples C, et al. Toward monitoring forest ecosystem integrity within the post‐2020 Global Biodiversity Framework. CONSERVATION LETTERS. 2021;14(4). doi: 10.1111/conl.12822

[42] CBD. Ecosystem Services Factsheet, Montreal, Canada; 2020. https://leap.unep.org/sites/default/files/2020-09/undb-factsheet-ecoserv-en.pdf

[43] WHO. WHO Constitution. Geneva, Switzerland; 1948. https://www.who.int/about/governance/constitution

[44] Faculty of Public Health. What is Public Health (webpage), London, UK; 2024. [cited 27 August 2024] https://www.fph.org.uk/what-is-public-health/#:~:text=is%20Public%20Health%3F-,Public%20health%20is%20the%20science%20and%20art%20of%20preventing%20disease,%2C%20leaving%20no%2Done%20behind

[45] IPCC. Sixth Assessment Report: Glossary. (webpage), Bonn, Germany; 2023. [cited 9 April 2025]. https://apps.ipcc.ch/glossary/

[46] WHO (no date). Environment, Climate Change, and Health - Monitoring: Methods for estimating health impacts. (webpage) [cited September 2024] https://www.who.int/teams/environment-climate-change-and-health/monitoring/methods

[47] Wilson EO, editor. Biodiversity. Washington, DC, USA: National Academy Press; 1988. ISBN: 0-309-56736-X, https://www.csu.edu/cerc/researchreports/documents/BiodiversityEOWilson1988.pdf

[48] Lajaunie C, Morand S. Biodiversity Targets, SDGs and Health: A New Turn after the Coronavirus Pandemic?. Sustainability. 2021;13(8):4353. doi: 10.3390/su13084353

[49] Gittleman JL. “One Health” needs ecology. Proc Natl Acad Sci U S A. 2024;121(50):e2413367121. doi: 10.1073/pnas.2413367121 39642201 PMC11648861

[50] Stewart-Ibarra A, LaBeaud AD. Transforming Global Health Partnerships. Switzerland: Springer Nature. 2024.

[51] U.S. National Academies of Science, Engineering, and Medicine. Integrating Public and Ecosystem Health Systems to Foster Resilience: A Workshop to Identify Research to Bridge the Knowledge-To-Action Gap, Washington, DC, USA and online; 2023. https://www.nationalacademies.org/our-work/integrating-public-and-ecosystem-health-systems-to-foster-resilience-a-workshop-to-identify-research-to-bridge-the-knowledge-to-action-gap.

[52] WWF. Living Planet Report 2024 – A System in Peril. WWF, Gland, Switzerland; 2024. https://www.livingplanetindex.org/.

[53] Richardson K, Steffen W, Lucht W, Bendtsen J, Cornell SE, Donges JF, et al. Earth beyond six of nine planetary boundaries. Sci Adv. 2023;9(37):eadh2458. doi: 10.1126/sciadv.adh2458 37703365 PMC10499318

[54] World Economic Forum. Global Risks Report 2024, 19th edition, Geneva, Switzerland; 2024. https://www.weforum.org/publications/global-risks-report-2024/in-full/global-risks-2034-over-the-limit/

[55] Niemeijer D, de Groot RS. A conceptual framework for selecting environmental indicator sets. Ecological Indicators. 2008;8(1):14–25. doi: 10.1016/j.ecolind.2006.11.012

[56] Persson L, Carney Almroth BM, Collins CD, Cornell S, de Wit CA, Diamond ML, et al. Outside the Safe Operating Space of the Planetary Boundary for Novel Entities. Environ Sci Technol. 2022;56(3):1510–21. doi: 10.1021/acs.est.1c04158 35038861 PMC8811958

[57] GEO BON. What are EESVs? (webpage) Quebec Centre for Biodiversity Science - McGill University, Canada; 2014. [cited April 14 2025] https://geobon.org/ebvs/ecosystem-services-working-group/what-are-eesvs/

[58] Belesova K, Haines A, Ranganathan J, Seddon J, Wilkinson P, Zou M. Designing a Planetary Health Watch: A system for integrated monitoring of the health effects of, and responses to, environmental change. Transdisciplinary Stakeholder Engagement, a workshop report. London, UK: Wellcome Trust; 2019. https://researchonline.lshtm.ac.uk/id/eprint/4654610/9/Belesova_etal_2019_Designing-a-Planetary-Health-Watch.pdf

[59] Romanello M, Walawender M, Hsu S-C, Moskeland A, Palmeiro-Silva Y, Scamman D, et al. The 2024 report of the Lancet Countdown on health and climate change: facing record-breaking threats from delayed action. Lancet. 2024;404(10465):1847–96. doi: 10.1016/S0140-6736(24)01822-1 39488222 PMC7616816

[60] UK Health Security Agency. Climate change and public health indicators: scoping review. 2023. [cited May 5 2025]. https://assets.publishing.service.gov.uk/media/64e87567635870000d1dbf6b/climate-change-and-public-health-indicators-scoping-review.pdf

[61] Hambling T, Weinstein P, Slaney D. A review of frameworks for developing environmental health indicators for climate change and health. Int J Environ Res Public Health. 2011;8(7):2854–75. doi: 10.3390/ijerph8072854 21845162 PMC3155333

[62] Chiabai A, Quiroga S, Martinez-Juarez P, Higgins S, Taylor T. The nexus between climate change, ecosystem services and human health: Towards a conceptual framework. Sci Total Environ. 2018;635:1191–204. doi: 10.1016/j.scitotenv.2018.03.323 29710574

[63] Murage P, Hajat S, Macintyre HL, Leonardi GS, Ratwatte P, Wehling H, et al. Indicators to support local public health to reduce the impacts of heat on health. Environ Int. 2024;183:108391. doi: 10.1016/j.envint.2023.108391 38118211

[64] Dos S Ribeiro C, van de Burgwal LHM, Regeer BJ. Overcoming challenges for designing and implementing the One Health approach: A systematic review of the literature. One Health. 2019;7:100085. doi: 10.1016/j.onehlt.2019.100085 31016220 PMC6475629

[65] Sibim AC, Chiba de Castro WA, Kmetiuk LB, Biondo AW. One Health Index applied to countries in South America. Front Public Health. 2024;12:1394118. doi: 10.3389/fpubh.2024.1394118 39440173 PMC11495393

[66] Plowright RK, Ahmed AN, Coulson T, Crowther TW, Ejotre I, Faust CL, et al. Ecological countermeasures to prevent pathogen spillover and subsequent pandemics. Nat Commun. 2024;15(1):2577. doi: 10.1038/s41467-024-46151-9 38531842 PMC10965931

[67] Plowright RK, Parrish CR, McCallum H, Hudson PJ, Ko AI, Graham AL, et al. Pathways to zoonotic spillover. Nat Rev Microbiol. 2017;15(8):502–10. doi: 10.1038/nrmicro.2017.45 28555073 PMC5791534

[68] Nishi M, Subramanian S, Gupta H. The Biodiversity–Health–Sustainability Nexus: Integrated Solutions from Landscapes & Seascapes. United Nations University, Institute for the Advanced Study of Sustainability. 2022; No 34. https://collections.unu.edu/eserv/UNU:8880/UNU-IAS-PB-No34-2022.pdf

[69] Burgess ND, Ali N, Bedford J, Bhola N, Brooks S, Cierna A, et al. Global Metrics for Terrestrial Biodiversity. Annual Review of Environment and Resources. 2024;49(1):673–709. doi: 10.1146/annurev-environ-121522-045106

[70] Rockström J, Gupta J, Qin D, Lade SJ, Abrams JF, Andersen LS, et al. Safe and just Earth system boundaries. Nature. 2023;619(7968):102–11. doi: 10.1038/s41586-023-06083-8 37258676 PMC10322705

[71] IPBES. Summary for Policymakers of the Thematic Assessment Report on the Interlinkages among Biodiversity, Water, Food and Health of the Intergovernmental Science-Policy Platform on Biodiversity and Ecosystem Services. McElwee, PD, Harrison, PA, van Huysen, TL, Alonso Roldán, V, Barrios, E, Dasgupta, P, et al, editors. IPBES secretariat, Bonn, Germany. 10.5281/zenodo.13850289

[72] Seddon N, Chausson A, Berry P, Girardin CAJ, Smith A, Turner B. Understanding the value and limits of nature-based solutions to climate change and other global challenges. Philos Trans R Soc Lond B Biol Sci. 2020;375(1794):20190120. doi: 10.1098/rstb.2019.0120 31983344 PMC7017763

[73] Bateman IJ, Mace GM. The natural capital framework for sustainably efficient and equitable decision making. Nat Sustain. 2020;3(10):776–83. doi: 10.1038/s41893-020-0552-3

[74] Pascual U, Balvanera P, Anderson CB, Chaplin-Kramer R, Christie M, González-Jiménez D, et al. Diverse values of nature for sustainability. Nature. 2023;620(7975):813–23. doi: 10.1038/s41586-023-06406-9 37558877 PMC10447232

[75] WHO. 14th General Programme of Work: A global health strategy for 2025-2028 - advancing equity and resilience in a turbulent world. Geneva; 2025. https://www.who.int/about/general-programme-of-work/fourteenth

[76] Fuller R, Landrigan PJ, Balakrishnan K, Bathan G, Bose-O’Reilly S, Brauer M, et al. Pollution and health: a progress update. Lancet Planet Health. 2022;6(6):e535–47. doi: 10.1016/S2542-5196(22)00090-0 35594895 PMC11995256

[77] WHO. Guidelines for drinking-water quality: Fourth edition incorporating the first addendum. Geneva; 2017. [cited April 13 2025] https://www.who.int/publications/i/item/9789241549950 28759192

[78] Hernández-Blanco M, Costanza R, Chen H, deGroot D, Jarvis D, Kubiszewski I, et al. Ecosystem health, ecosystem services, and the well-being of humans and the rest of nature. Glob Chang Biol. 2022;28(17):5027–40. doi: 10.1111/gcb.16281 35621920

[79] Haahtela T. A biodiversity hypothesis. Allergy. 2019;74(8):1445–56. doi: 10.1111/all.13763 30835837

[80] Johnson PTJ, Thieltges DW. Diversity, decoys and the dilution effect: how ecological communities affect disease risk. J Exp Biol. 2010;213(6):961–70. doi: 10.1242/jeb.037721 20190121

[81] Civitello DJ, Cohen J, Fatima H, Halstead NT, Liriano J, McMahon TA, et al. Biodiversity inhibits parasites: Broad evidence for the dilution effect. Proc Natl Acad Sci U S A. 2015;112(28):8667–71. doi: 10.1073/pnas.1506279112 26069208 PMC4507196

[82] Natural England. A narrative review of reviews of nature exposure and human health and well-being in the UK. Natural England Evidence Review NEER030. 2024. http://www.gov.uk/natural-england

[83] Salkeld DJ, Padgett KA, Jones JH. A meta-analysis suggesting that the relationship between biodiversity and risk of zoonotic pathogen transmission is idiosyncratic. Ecol Lett. 2013;16(5):679–86. doi: 10.1111/ele.12101 23489376 PMC7163739

[84] Wood CL, McInturff A, Young HS, Kim D, Lafferty KD. Human infectious disease burdens decrease with urbanization but not with biodiversity. Philos Trans R Soc Lond B Biol Sci. 2017;372(1722):20160122. doi: 10.1098/rstb.2016.0122 28438911 PMC5413870

[85] Gluckman PD, Hanson MA. Developmental origins of disease paradigm: a mechanistic and evolutionary perspective. Pediatr Res. 2004;56(3):311–7. doi: 10.1203/01.PDR.0000135998.08025.FB 15240866

[86] CBD. Notification 2025-049: Extension of Deadline: Invitation to submit information for the development of indicators, metrics and progress measurement tools on Biodiversity and Health.(webpage and email); 2025. https://www.cbd.int/notifications/2025-049

[87] CBD. Report of the Ad hoc Technical Expert Group on Indicators for the Strategic Plan for Biodiversity 2011-2020 (UNEP/CBD/ID/AHTEG/2015/1/3, UNEP/CBD/SBSTTA/19/INF/5), 14-17 September, Geneva, Switzerland; 2015, and 19^th^ Meeting of the Subsidiary Body on Scientific, Technical, and Technological Advice, 2–5 November, Montreal, Canada; 2015. https://www.cbd.int/meetings/SBSTTA-19

[88] CBD. Decision X/2 The Strategic Plan for Biodiversity 2011-2020 and the Aichi Biodiversity Targets, 10^th^ Conference of the Parties to the Convention on Biological Diversity, 18-29 October, Nagoya, Japan; 2010. https://www.cbd.int/decision/cop?id=12268

[89] Maney C, Guaras D, Harrison J, Guizar-Coutiño A, Harfoot MBJ, Hill SLL, et al. National commitments to Aichi Targets and their implications for monitoring the Kunming-Montreal Global Biodiversity Framework. NPJ Biodivers. 2024;3(1):6. doi: 10.1038/s44185-024-00039-5 39242842 PMC11332214

[90] Willetts L, Siege C, Stewart-Ibarra AM, Horn O, Chotthong B, Tanawat T, et al. Advancing integrated governance for health through national biodiversity strategies and action plans. Lancet. 2023;402(10404):753–6. doi: 10.1016/S0140-6736(23)01431-9 37499672

[91] Whitehorn PR, Navarro LM, Schröter M, Fernandez M, Rotllan-Puig X, Marques A. Mainstreaming biodiversity: A review of national strategies. Biol Conserv. 2019;235:157–63. doi: 10.1016/j.biocon.2019.04.016 32218608 PMC7083249

[92] CBD. Note by the Executive Secretary: update on progress on revising/updating and implementing national biodiversity strategies and action plans, including national targets. 14th Conference of the Parties to the Convention on Biological Diversity, Sharm El-Sheikh, Egypt; 2018. https://www.cbd.int/doc/c/3d50/c310/2e8a0f5f3b44fd8c0df5f7f3/cop-14-05-add1-en.pdf (accessed April 13, 2025)

[93] CBD. Decision 16/31 Monitoring framework for the Kunming-Montreal Global Biodiversity Framework, 16^th^ Conference of the Parties to the Convention on Biological Diversity, second resumed session, Rome, Italy; 2025. https://www.cbd.int/doc/decisions/cop-16/cop-16-dec-31-en.pdf

[94] CBD. Guidance on using the indicators of the monitoring framework of the Kunming-Montreal Global Biodiversity Framework (CBD/SBSTTA/26/INF/14), 26^th^ Meeting of the Subsidiary Body on Scientific, Technical, and Technological Advice, 13–18 May, Nairobi, Kenya; 2024. https://www.cbd.int/doc/c/92cf/b458/18519b4c0b487bf9bfc23988/sbstta-26-inf-14-en.pdf

[95] CBD. Monitoring framework for the Kunming-Montreal Global Biodiversity Framework, Recommendation to the 16th Conference of the Parties (CBD/SBSTTA/REC/26/1), 26th Meeting of the Subsidiary Body on Scientific, Technical, and Technological Advice, 13-18 May, Nairobi, Kenya; 2024 https://www.cbd.int/doc/recommendations/sbstta-26/sbstta-26-rec-01-en.pdf

[96] Brauman KA, Garibaldi LA, Polasky S, Zayas CN, Aumeeruddy Thomas Y, Brancalion P. Status and trends—nature’s contributions to people (NCP). In: Brondizio ES, Settele SD, Ngo HT, editors. Global assessment report on biodiversity and ecosystem services. Bonn, Germany: IPBES. 2019. doi: 10.5281/zenodo.3831673

[97] Willetts L, van de Pas R, Woolaston K, Bennett NJ, Vora NM, Shah D, et al. Implementing the Global Action Plan on Biodiversity and Health. Lancet. 2024;404(10470):2402–5. doi: 10.1016/S0140-6736(24)02557-1 39615507

[98] IPBES. Summary for Policymakers of the Thematic Assessment Report on the Underlying Causes of Biodiversity Loss and the Determinants of Transformative Change and Options for Achieving the 2050 Vision for Biodiversity. O’Brien, K, Garibaldi, L, Agrawal, A, Bennett, E, Biggs, R, Calderón Contreras, R, editors. IPBES secretariat, Bonn, Germany. 2025; doi: 10.5281/zenodo.11382230

[99] CBD. Decision 15/6 Mechanisms for planning, monitoring, reporting and review (CBD/COP/DEC/15/6), 15th Conference of the Parties Part II, 7-19 December, Montreal, Canada; 2022. https://www.cbd.int/decisions/cop?m=cop-15

[100] CBD. Mechanisms for planning, monitoring, reporting and review (SBI/4/4), Note by the Secretariat, 4^th^ Meeting of the Subsidiary Body on Implementation, 21–29 May Nairobi, Kenya; 2024. https://www.cbd.int/doc/c/00f6/998f/a319de28094f1ace03609c5f/sbi-04-04-en.pdf

[101] UNPFII. Note by the Secretariat: Improving the health and wellness of Indigenous Peoples globally: operationalization of Indigenous determinants of health (E/C.19/2024/5), 23^rd^ Session of the UN Permanent Forum on Indigenous Issues, New York, NY, USA; 2024. https://social.desa.un.org/sites/default/files/EN_0.pdf

[102] Redvers N, Poelina A, Schultz C, Kobei DM, Githaiga C, Perdrisat M, et al. Indigenous Natural and First Law in Planetary Health. Challenges. 2020;11(2):29. doi: 10.3390/challe11020029

[103] Boyd F, Allen C, Robinson JM, Redvers N. The past, present, and future of nature and place-based interventions for human health. Landscape Research. 2023;49(1):129–45. doi: 10.1080/01426397.2023.2244430

[104] Heke I. Atua Matua Framework. [cited 2024 September 10] https://www.atuamatua.co.nz/atua-matua

[105] CBD. Decision 15/5 Monitoring framework for the Kunming-Montreal Global Biodiversity Framework (CBD/COP/DEC/15/5), 15th Conference of the Parties Part II, 7-19 December, Montreal, Canada; 2022. https://www.cbd.int/decisions/cop?m=cop-15

[106] CBD. Decision 15/22 Nature and Culture, 15th Conference of the Parties Part II CBD/COP/DEC/15/22), 7-19 December, Montreal, Canada; 2022. https://www.cbd.int/decisions/cop?m=cop-15

[107] CBD. Scientific and technical review of the traditional knowledge indicators and their suggested links with the headline, component and complementary indicators of the monitoring framework for the Kunming-Montreal Global Biodiversity Framework (SBSTTA 26/INF/11), 26^th^ Meeting of the Subsidiary Body on Scientific, Technical, Technological Advice, 13-18 May, Nairobi, Kenya; 2024.

[108] Baum SE, Machalaba C, Daszak P, Salerno RH, Karesh WB. Evaluating one health: Are we demonstrating effectiveness?. One Health. 2016;3:5–10. doi: 10.1016/j.onehlt.2016.10.004 28616496 PMC5458598

[109] World Bank. Putting Pandemics Behind Us: Investing in One Health to Reduce Risks of Emerging Infectious Diseases. Washington, DC; 2022. https://openknowledge.worldbank.org/entities/publication/b8ac824f-1693-5226-b3bf-4d634f5e869e

[110] Zhou X-N, Guo X, Zhang X. Global One Health Index Report 2022. Springer Nature Singapore. 2025. doi: 10.1007/978-981-97-4824-2

[111] WHO, FAO of the UN, UNEP and WOAH. A guide to implementing the One Health Joint Plan of Action at national level. Geneva, Switzerland; 2023. https://wedocs.unep.org/bitstream/handle/20.500.11822/44353/one_health_joint_plan.pdf?sequence=1&isAllowed=y

[112] WHO. Template for a National One Health Work Plan. Geneva, Switzerland; 2023. https://www.who.int/publications/m/item/template-for-a-national-one-health-workplan

[113] UNEP and International Science Council. Navigating New Horizons: A global foresight report on planetary health and human wellbeing. Nairobi, Kenya; 2024. https://doi.og/10.59117/20.500.11822/45890

[114] IPCC. Climate Change 2022: Impacts, Adaptation, and Vulnerability. Contribution of Working Group II to the Sixth Assessment Report of the Intergovernmental Panel on Climate Change. Pörtner, H-O, Roberts, DC, Tignor, MMB, Poloczanska, E, Mintenbeck, K, Alegría, A, et al, editors. Cambridge, UK: Cambridge University Press; 2022. https://www.ipcc.ch/report/ar6/wg2/

[115] Brousselle A, Curren M, Dunbar B, McDavid J, Logtenberg R, Ney T. Planetary health: Creating rapid impact assessment tools. Evaluation. 2024;30(2):269–87. doi: 10.1177/13563890241227433

[116] Stockholm Resilience Center. (webpage) [cited 27 August 2024] https://www.stockholmresilience.org/research/planetary-boundaries.html

[117] WHO. Preventing disease through healthy environments: a global assessment of the burden of disease from environmental risks. Geneva, Switzerland; 2016. https://www.who.int/publications/i/item/9789241565196

[118] CBD. Biodiversity and health, Note by the Secretariat [CBD/SBSTTA/26/8]. 26^th^ Meeting of the Subsidiary Body on Scientific, Technical and Technological Advice, 13–18 May, Nairobi, Kenya; 2024. https://www.cbd.int/doc/c/ac0e/89c0/0fd5151bf882430aee708179/sbstta-26-08-en.pdf

[119] CBD. Biodiversity and Health, Note by the Executive Secretary (CBD/SBSTTA/24/9), 24^th^ Meeting of the Subsidiary Body on Scientific, Technical, and Technological Advice, 3 May – 13 June, Online; 2021. https://www.cbd.int/doc/c/76f9/1b75/42e360ab3ae6e53d0762c449/sbstta-24-09-en.pdf

[120] UNFCCC. Decision 2/CMA.5 Global Goal on Adaptation (FCCC/PA/CMA/2023/16/Add.1), 28^th^ Conference of the Parties of the UNFCCC, 30 November-12 December, Dubai, United Arab Emirates; 2023 https://unfccc.int/event/cma-5#decisions_reports

[121] UNEP. Global Framework on Chemicals: for a Planet Free of Harm from Chemicals and Waste, 5th International Conference on Chemicals Management (ICCM5), 25-29 September, Bonn, Germany; 2023 https://www.unep.org/resources/global-framework-chemicals-planet-free-harm-chemicals-and-waste

[122] UN Office for Disaster Risk Reduction. Sendai Framework for Disaster Risk Reduction 2015-2030. [cited April 7 2025]. https://www.undrr.org/media/16176/download?startDownload=20250417

[123] United Nations University Institute for Environment and Human Security. Interconnected Disaster Risks: Turning Over a New Leaf. Eberle, C, Narvaez, L, Mena Benavides, M, Karakislak, I, Sebesvari, Z, authors. Bonn: United Nations University Institute for Environment and Human Security. 2025; doi: 10.53324/AZOO7042

[124] CBD. Decision 16/6. Role of people of African descent, comprising collectives embodying traditional lifestyles, in the implementation of the Convention on Biological Diversity, 16th Conference of the Parties to the Convention on Biological Diversity, 21 October-1 November, Calí, Colombia; 2024. https://www.cbd.int/doc/decisions/cop-16/cop-16-dec-06-en.pdf [cited June 17 2025]

[125] WHO. WHO Policy Brief: Loss and Damage. Geneva, Switzerland; 2022. https://www.who.int/publications/m/item/who-policy-brief--loss-and-damage

[126] Loss and Damage Youth Coalition. Non-Economic Loss and Damage (NELD) Understanding and Addressing Response Gaps: Policy Brief. International Institute for Environment and Development; 2023. https://weadapt.org/wp-content/uploads/2023/12/LDYC-Policy-Brief.pdf

[127] Willetts L. Planetary Health and Disaster Risk Reduction: the Sendai Framework at its Midpoint. Lancet Planet Health. 2024;8(9):e613–5. doi: 10.1016/S2542-5196(24)00200-6 39146943

[128] BRS and Minamata. Interlinkages between the chemicals and waste multilateral environmental agreements and biodiversity: Key insights. Geneva, Switzerland; 2021. https://www.brsmeas.org/Implementation/InterlinkageswithotherMEAsandprocesses/ConventiononBiologicalDiversity/Publications/tabid/9889/language/en-US/Default.aspx#.

[129] WHO. 14^th^ General Programme of Work, 2025-2028 (WHA77.1), 77^th^ Session of the World Health Assembly, 27 May – 1 June, Geneva, Switzerland; 2024. https://apps.who.int/gb/ebwha/pdf_files/WHA77/A77_R1-en.pdf

[130] UNEP. Resolution 6/4 Promoting synergies, cooperation or collaboration for national implementation of multilateral environ (UNEP/EA.6/Res.4), 6^th^ UN Environment Assembly, 26 February–1 March, Nairobi, Kenya; 2024. https://wedocs.unep.org/handle/20.500.11822/45383

