# Supplementary material for: Metrics for biodiversity and health policy integration
Source: PLOS Glob Public Health. 2025 Jul 1;5(7):e0004624. doi: 10.1371/journal.pgph.0004624 (PMC12212572; doi:10.1371/journal.pgph.0004624)
Supplement: S1 Table — (DOCX) [file pgph.0004624.s001.docx]

| **S1 Table**  **Intergovernmental Req﻿uests for Biodiversity-Health Indicators**  **Under the﻿ Convention on Biological Diversity Between 2012 and 2024** | | | |
| --- | --- | --- | --- |
| Date | Event | Document | Consensus Requests from COPs |
| 2012 | COP 11 | Decision XI/6 Cooperation with other conventions, international organizations, and initiatives | Requests the Secretariat, in collaboration with relevant organizations and based on the views of Parties, to **develop Aichi Target indicators** **relevant to biodiversity-health interlinkages,** including trends in benefits that humans derive from selected ecosystem services, trends in health and well-being of communities that depend directly on local ecosystem goods and services, and trends in the nutritional contribution of biodiversity and food composition, and encourages Parties, other Governments and relevant stakeholders, to make use of them |
| 2014 | COP 12 | Decision XII/21 Biodiversity and human health | Requests the CBD Secretariat subject to the availability of resources to **develop** **indicators on biodiversity and human health** |
| 2016 | COP 13 | Decision XIII/6 Biodiversity and human health | encouraged parties, governments, and other relevant organizations to “**develop** **integrated metrics, indicators and tools** to facilitate the analysis, evaluation, monitoring and integration of biodiversity into health strategies, plans and programmes and vice-versa” as well as to compile **toolkits** |
| 2018 | COP 14 | Decision 14/4  Health and Biodiversity | Requests the Executive Secretary, subject to the availability of financial resources, and invites the World Health Organization, in collaboration, as appropriate, with other members of the Interliaison Group on Biodiversity and Health to **develop integrated science-based indicators, metrics and progress measurements tools** on biodiversity and health |
| 2022 | COP 15 | Decision 15/29  Biodiversity and Health | Invites the Quadripartite for One Health, the One Health High-Level Expert Panel, and other relevant expert groups and initiatives to contribute to the **development of, and reporting on,** **health-related indicators of the monitoring framework for the Kunming-Montreal Global Biodiversity Framework** |
| 2024 | COP  16 | Decision 16/19 Biodiversity and Health | Requests the Executive Secretary, subject to the availability of resources **to complete the work of decision 14/4 on the development of integrated science-based indicators, metrics and progress measurement tools** on biodiversity and health  Voluntarily, governments and other actors are encouraged **to integrate biodiversity-related metrics, indicators and tools into health strategies**, plans and programmes and, conversely**, the integration of health-related metrics, indicators and tools into biodiversity strategies**, plans and programmes; and explore the possibilities of developing indicators on linkages between climate, biodiversity and health. |
| Source: Convention on Biological Diversity website, <https://www.cbd.int/decisions> (accessed May 8 2025)  COP – conference of the parties; CBD – Convention on Biological Diversity  Citation: Nogués-Bravo, Whitmee, and Willetts (2025) Metrics for biodiversity and health policy integration. PLOS Glob Public Health 5(6): e0004624. https://doi.org/10.1371/journal.pgph.0004624 | | | |

**Supporting Information**
